# Sortase-Modified Cholera Toxoids Show Specific Golgi Localization

**DOI:** 10.3390/toxins16040194

**Published:** 2024-04-16

**Authors:** Darren C. Machin, Daniel J. Williamson, Peter Fisher, Victoria J. Miller, Zoe L. P. Arnott, Charlotte M. E. Stevenson, Gemma C. Wildsmith, James F. Ross, Christopher W. Wasson, Andrew Macdonald, Benjamin I. Andrews, Daniel Ungar, W. Bruce Turnbull, Michael E. Webb

**Affiliations:** 1 School of Chemistry and Astbury Centre for Structural Molecular Biology, University of Leeds, Leeds LS2 9JT, UK; d.c.machin@leeds.ac.uk (D.C.M.);; 2 Department of Biology, University of York, York YO10 5DD, UK; 3 Department of Biochemistry, University of Bristol, Bristol BS8 1QU, UK; 4 Faculty of Biological Sciences, Astbury Centre for Structural Molecular Biology, University of Leeds, Leeds LS2 9JT, UKa.macdonald@leeds.ac.uk (A.M.); 5 GlaxoSmithKline, Medicines Research Centre, Gunnels Wood Road, Stevenage SG1 2NY, UK

**Keywords:** cholera toxin, sortase, protein labeling, cellular imaging, Golgi body

## Abstract

Cholera toxoid is an established tool for use in cellular tracing in neuroscience and cell biology. We use a sortase labeling approach to generate site-specific N-terminally modified variants of both the A2-B_5_ heterohexamer and B_5_ pentamer forms of the toxoid. Both forms of the toxoid are endocytosed by GM1-positive mammalian cells, and while the heterohexameric toxoid was principally localized in the ER, the B_5_ pentamer showed an unexpectedly specific localization in the medial/trans-Golgi. This study suggests a future role for specifically labeled cholera toxoids in live-cell imaging beyond their current applications in neuronal tracing and labeling of lipid rafts in fixed cells.

## 1. Introduction

**Figure 1:**
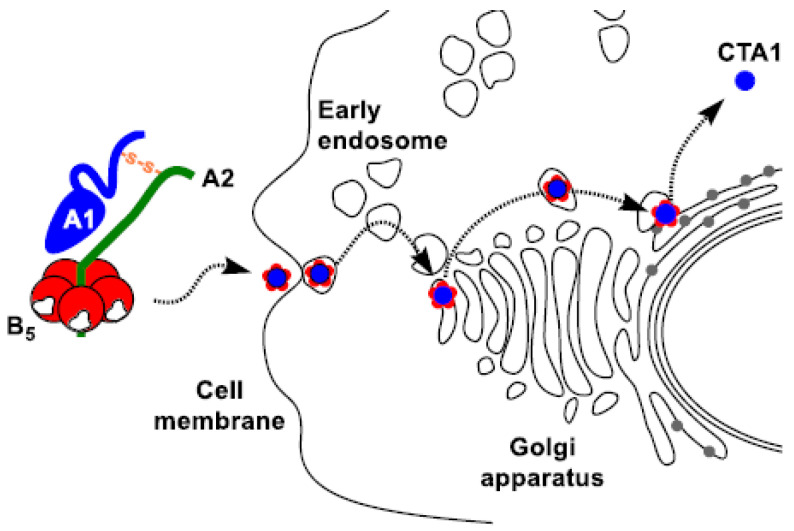
Cell entry mechanism for intact cholera toxin showing transit of the holotoxin through the cell (arrows). Binding to the cell surface is mediated by interaction of the B-subunits with ganglioside GM1, which induces endocytosis. The toxin is trafficked via the trans-Golgi network to the ER, mediated by interaction of the KDEL sequence on the A2 peptide with the KDEL receptor. Following disulfide cleavage, the partially unfolded A1 subunit is a substrate for the ER-associated degradation pathway leading to transport into the cytosol.

Cholera toxin and the closely related heat-labile enterotoxin share an identical hexameric AB_5_ structure composed of five B-subunits (B_5_) arranged in a pentamer around a central A-subunit (Figure 1) [1,2,3]. The B_5_ pentamer is a carbohydrate-binding protein that can bind ganglioside [GM1] [4,5,6] and fucosylated glycans [7,8,9] on the surface of mammalian cells, triggering endocytosis of the toxin via one or more of at least six distinct endocytic mechanisms [10]. While the pentamer has five binding sites for GM1, only one is sufficient to enable cell entry and intoxication [4,11]. Once internalized, the protein undergoes cytoskeleton-dependent retrograde trafficking [12] via the trans-Golgi network (TGN) to the endoplasmic reticulum (ER), directed by the interaction of the membrane-bound KDEL receptor with a short KDEL targeting sequence located at the C-terminus of the A-subunit [13,14,15,16]. The peptide chain between the A1 and A2 subunit is cleaved in the TGN, and upon arrival in the ER, the A1-domain is released from the rest of the toxin by protein disulfide isomerase (PDI) [17,18]. This release leads to partial unfolding of the A2 domain, leading to retrotranslocation into the cytosol [19,20,21]. ADP-ribosylation factor-6 binds to the A1-protein in the cytosol, allowing it to reach its target destination, G-protein, and induce toxicity via the activation of adenylyl cyclase [22,23]. Importantly, the retrograde trafficking mechanism used by the bacterial toxins to gain entry into cells is not dependent upon either the catalytic A1-domain or A-subunit but is rather dependent upon the binding of the B-subunit.

The intrinsic ability of bacterial toxins to induce endocytosis has been exploited in several studies to transport biomolecules and probes into mammalian cells both in vivo and in vitro [24,25,26,27,28,29,30]. Intracellular delivery has predominantly been achieved through the use of bacterial toxin chimeras [29,31,32,33], but this approach requires a gene construct to be created for every novel protein fusion, and it is quite likely that expression optimization would also be necessary. Guimares et al. used sortase A (SrtA) to attach a selection of chemical probes and a protein to the A1-domain of a cholera toxin analog, allowing the intracellular fate of the protein to be studied in more detail [34]. In this case, a recombinant holotoxin containing an LPETG recognition motif for SrtA and a trypsin cleavage site between the A1 and A2 domains was cleaved with trypsin before the C-terminal labeling of the A1 domain. However, these molecules retain toxic action, so their application as a general delivery system is limited. In a separate study, the N-terminus of the B-subunit was also labeled using SrtA-mediated ligation, but the ability of the modified proteins to form stable pentamers and enter mammalian cells was not established [35]. The binding of CTB to the cell surface induces clustering of GM1 lipids and membrane curvature, which is thought to be linked to cell entry, and it has, therefore, been used extensively as a probe of lipid raft formation in fixed cell imaging [36,37]. Despite this, relatively few studies of its individual localization have been undertaken [38].

SrtA is a type II membrane-bound protein that “sorts” and covalently anchors virulence factors to the cell wall of Gram-positive bacteria [39,40,41,42]. The enzyme binds to proteins carrying an LPXTG recognition motif and attaches them to peptidoglycans bearing an N-terminal oligoglycine sequence. This ligation mechanism has been used in numerous studies [43,44,45,46] to label the N- and/or C-terminus of proteins with a range of chemical probes, including biotin, fluorescein, and cholesterol. The advantage of SrtA-mediated ligation over other protein labeling techniques is that it only requires the introduction of a single sterically accessible glycine residue at the N-terminus of a protein substrate or an LPXTGX motif at the C-terminus. A wide range of studies have explored enhancing the activity [47,48,49] and changing the specificity of this enzyme [50,51,52]. In addition, a variety of strategies have been reported to enable more efficient reactions [53,54,55], including the use of depsipeptide substrates, which can greatly increase the N-terminal labeling efficiency [56]. In this work we sought to implement this optimized strategy to generate both nontoxic A2-B_5_ and B_5_ toxoids and to confirm the localization of these proteins in mammalian cells.

## 2. Results and Discussion

### 2.1. Labeling and Localization of an A2-B_5_ Toxoid Construct

**Figure 2:**
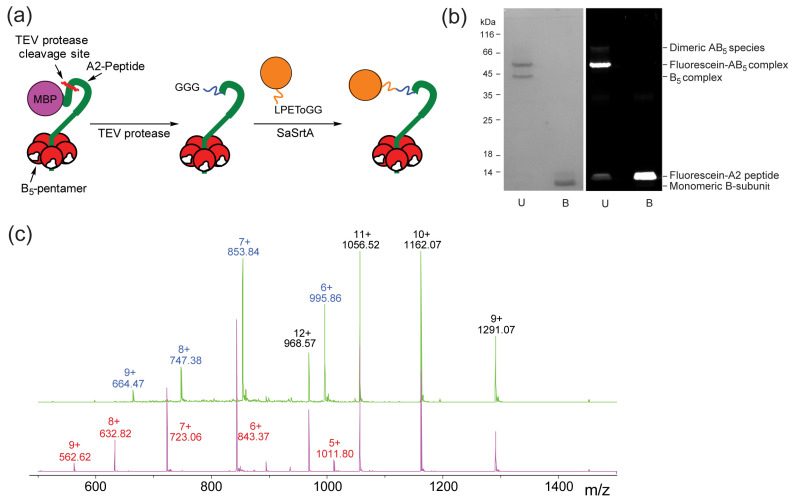
Strategy and results for single labeling of ChTx A2-B_5_ complex. (**a**) The MBP-A2-B_5_ construct enables convenient double affinity purification of the intact A2-B_5_ complex prior to TEV cleavage to reveal an N-terminal glycine for sortase labeling. (**b**) SDS-PAGE analysis of sortase-labeled protein after purification with and without boiling. (**c**) ES-MS analysis of the A2-B_5_ protein before (upper spectrum) and after sortase labeling shows near quantitative transformation of the A2 peptide into the sortase-labeled A2 form.

We took two approaches to generate modified cholera toxoids, based either on single labeling of a truncated A2-B_5_ toxoid or multiple functionalizations of the B_5_ subunit alone. For the A2-B_5_ cholera toxoid, we modified existing constructs for production of biotinylated AB_5_ proteins by oxime formation [57,58] and generated an expression construct in which the coding sequence for a maltose binding protein-triglycine-A2 fusion protein was 5′ to that for the B-subunit in a polycistronic construct under the control of the lac promoter in an adapted pMalc5x backbone (see supplementary information). The expressed proteins are both co-translationally exported into the periplasm before assembly and export into the growth medium. While traces of the fusion protein could be detected in both the periplasm and the growth medium, we isolated it from the growth medium via ammonium sulfate precipitation followed by sequential amylose and immobilized metal affinity chromatography to yield the protein complex. Treatment of the MBP-A2-B_5_ construct with TEV protease yielded an N-terminal triglycine motif on the A2 subunit. Using our reported sortase labeling approach, after test labeling using a dansyl peptide (see supplementary information), the A2-B_5_ complex (43 μM) was quantitatively labeled at 37 °C using a two-fold excess of a FITC-depsipeptide (90 μM) and 12 mol% sortase (Figure 2a) followed by purification by size-exclusion chromatography. If the samples are not boiled, both the B_5_ pentamer and the A2-B_5_ complex are sufficiently stable to electrophorese as multimers in SDS-PAGE (Figure 2b). Unlabeled protein is not observed in the SDS-PAGE analysis, and although trace peaks corresponding to the unlabeled protein (Figure 2c) can be detected via ES-MS, these are similar in intensity to other minor contaminating species.

**Figure 3:**
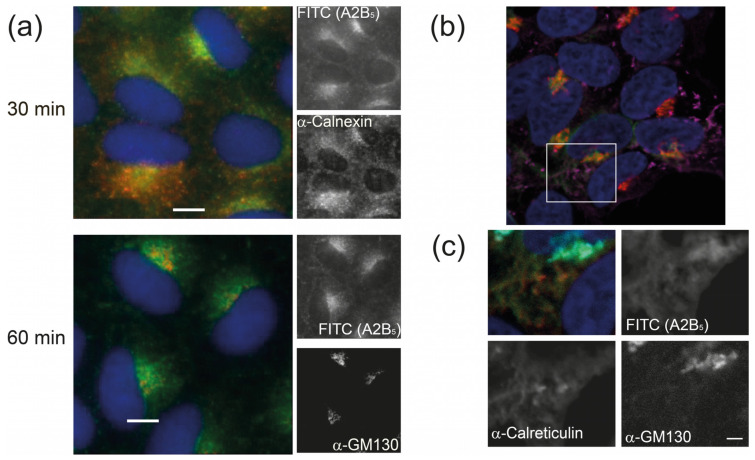
Sortase-labeled ChTx A2-B_5_ is found in both the ER and Golgi. (**a**) B-SC-1 cells incubated with FITC-A2-B_5_ (green) for the indicated times, stained following fixation for calnexin (red, top) or GM130 (red, bottom). Scale bar 10 μM (**b**) HEK293 Cells incubated with FITC-A2-B_5_ (green) for 5 min before incubation in fresh growth media for 2.5 h, followed by fixation and staining for calreticulin (purple) and GM130 (red). (**c**) Close-up of inset region in (**b**) showing distribution of A2-B5 between the Golgi and ER. FITC-A2-B_5_ (green), α-Calreticulin (red), α-GM130 cyan. Individual channels are shown in black and white. Scale bar 2 μM.

The trafficking and localization pattern of the FITC-A2-B_5_ was initially studied in monkey epithelial kidney (B-SC-1) cells. The cells were incubated with 150 nM A2-B_5_ complex in medium and before acid washing to remove surface-bound proteins, fixing with MeOH, counterstaining with antibodies to calnexin (ER) and GM130 (*cis*-Golgi) marker proteins and wide-field fluorescence imaging (Figure 3a). After 5 min of uptake, the protein was largely observed in small punctate bodies consistent with endocytic vesicles. After 30 min, the protein was located diffusely throughout the cell with some concentration in a perinuclear body consistent with localization in both the ER (shown by colocalization with the calnexin marker) and the Golgi. After 60 min, the protein was principally concentrated in a perinuclear location consistent with the Golgi, adjacent to but not coincident with the GM130 *cis*-Golgi marker. This observation of trafficking to the ER and to Golgi-associated membrane bodies is consistent with previous observations of cholera toxin trafficking. The observation of the protein largely in a Golgi-associated compartment after an hour rather than in the ER was unexpected. We, therefore, repeated the experiment (in HEK293 cells, Figure 3b) using a pulse-chase approach in which the A2-B_5_ toxin was initially added to the cells before washing and a further 2.5 h incubation in a growth medium (Figure 3b). In this case, a mixed Golgi/ER localization is observed. This suggests that the capacity of the cell to traffic the A2-B_5_ from the cell surface to the Golgi is substantially greater than the capacity of its KDEL-linked transport to the ER.

### 2.2. Labeling of the B_5_ Toxoid

**Figure 4:**
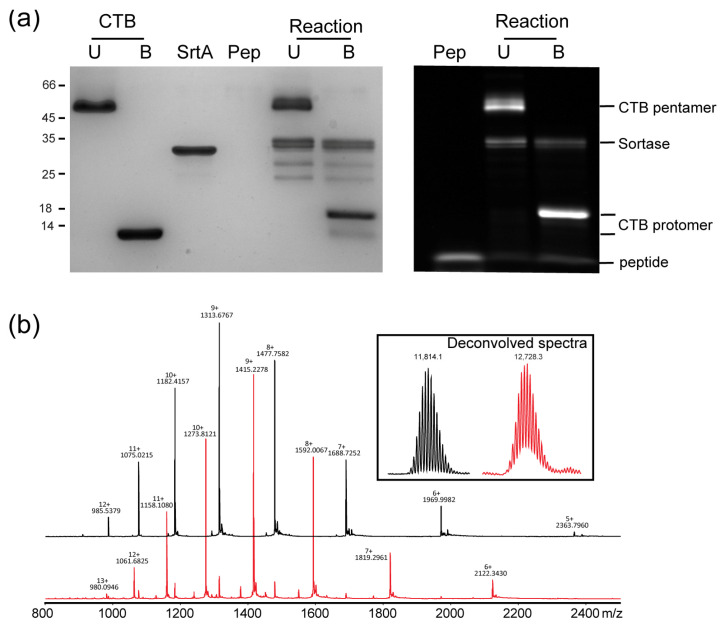
Labeling of CTB to form FITC-CTB. (**a**) Labeling of CTB using WT-SrtA. Following labeling, a fluorescent band is observed for the CTB pentamer, but the labeled pentamer is not resolved from the unlabeled pentamer; the boiled sample indicates the presence of a small amount of unlabeled monomer. Labeling of CTB is accompanied by labeling of the SrtA construct, which also has an N-terminal glycine. (**b**) ES-MS analysis of unlabeled CTB (black) and CTB after labeling reaction (red). Inset: deconvolved mass spectra confirm expected mass for unlabeled and labeled protein (unlabeled: observed 11,814.1, expected 11,814; labeled: observed 12,728.3, expected). Level of labeling can be estimated at ~90% based on intensity of bands in Coomassie-stained SDS-PAGE, relative intensity of bands for matched charge states (e.g., 10^+^ 1182.4 and 1273.8) or from deconvolved spectrum.

Following the labeling of the A2-B_5_ system, we sought to use the same approach to modify the simpler cholera toxin B homopentamer to determine whether we would observe the same transport to the Golgi. Naturally occurring cholera toxoids have either an N-terminal alanine or threonine. While the latter isoform can be labeled using periodate-mediated oxidation and imine formation [59], we wished to use the more chemically stable peptide formed as a result of labeling using sortase. Ploegh and coworkers have previously demonstrated that a triglycine extension is required for efficient labeling of this scaffold [35]. We, therefore, generated cholera toxin B with an N-terminal triglycine extension (GGG-CTB). Briefly, the coding sequence was generated using an optimized protocol for assembly PCR and subcloned into a variant of pMALp5x in which the *malE* gene has been replaced with the LTB-leader sequence for periplasmic expression. Protein was overexpressed in *E. coli* C41 (DE3) cells and purified from the growth medium by sequential ammonium sulfate precipitation, immobilized metal-affinity chromatography and size-exclusion chromatography. Labeling of the protein (120 μM protomer concentration) was carried out by incubation for three hours with three equivalents of a FITC-depsipeptide (360 μM) and 20 mol% sortase at 37 °C to generate FITC-CTB (Figure 4). Analysis of the labeling reaction by SDS-PAGE and ES-MS indicated >90% labeling of the protein. FITC-CTB was isolated from excess peptide and sortase by size-exclusion chromatography.

**Figure 5:**
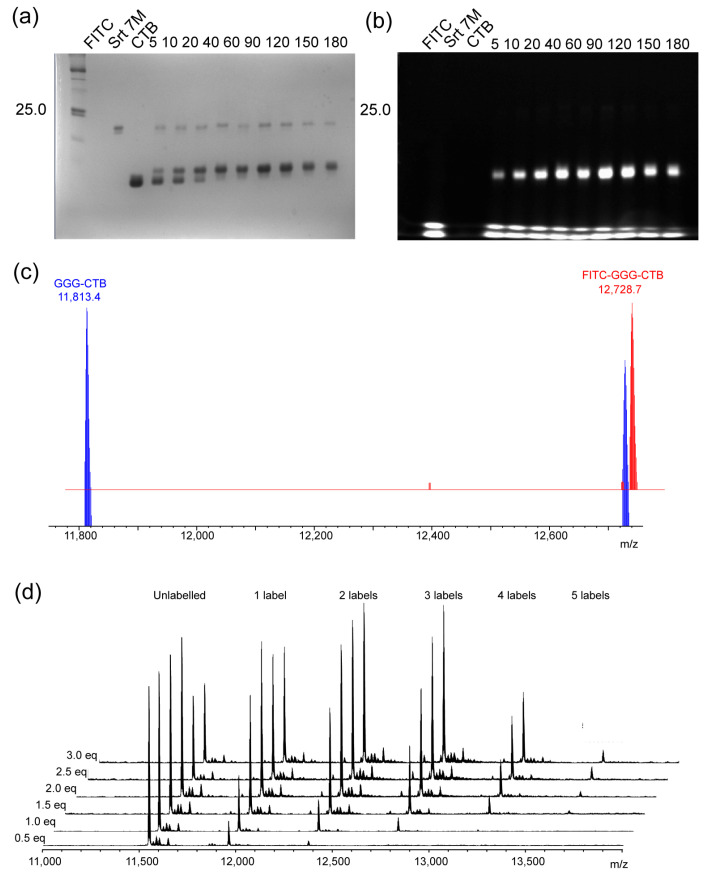
Labeling of CTB to form FITC-CTB and TAMRA-CTB. Labeling of CTB using Srt7M. (**a**,**b**) Analysis of Srt7M/depsipeptide-mediated labeling of CTB via SDS-PAGE. CTB (50 μM) using Srt7M (2.5 mM) and depsipeptide (150 mM) in 50 mM HEPES, 150 mM NaCl pH 7.5. (**c**) ES-MS analysis of the labeling reaction at 1 h (blue) and 2 h (red). Labeling of the Srt7M is not observed due to the absence of an N-terminal glycine in this construct. (**d**) ES-MS analysis of an exemplar NHS ester. CTB H74A (250 mM) was incubated with TAMRA-NHS ester (noted relative concentration) for 1 h in 50 mM CHES pH 9.3, 50 mM NaCl before analysis by ESMS.

Labeling of CTB using WT-SrtA gave only ~90% labeling based on the ratio of bands as compared to the quantitative labeling observed for the A2-B_5_ peptide (Figure 4). We attributed this to the balance between the labeling reaction and hydrolytic reaction on the pentameric scaffold in the presence of a relatively large amount of catalyst (20%) as well as the propensity of the catalyst itself to be labeled due to the presence of an N-terminal glycine in our expression construct. We, therefore, investigated the effect of using the evolved Ca-independent Srt7M mutant [49] in which a lower concentration of sortase could, in theory, be used and the expression construct for which does not encode an N-terminal glycine. It was possible to label CTB quantitatively using 5 mol% (2.5 μM) catalyst to label 50 μM protein with a 3-fold excess of depsipeptide, and the reaction was followed by SDS-PAGE and ESMS (Figure 5a–c). Finally, we wanted to compare the labeling of CTB with more conventional approaches to labeling. CTB is commercially available and non-specifically labeled with fluorophores. We, therefore, investigated the specificity of such labeling using an available CTB site-directed mutant (CTB H94A) and investigated the extent to which it could be controlled by varying the concentration of the labeling reagent (Figure 5d). Up to five labeling events per protomer could be detected, and even when using 0.5 equivalents, a mixture of unlabeled, singly-labeled and doubly-labeled proteins was still observed.

### 2.3. Localization of the B_5_ Toxoid

**Figure 6:**
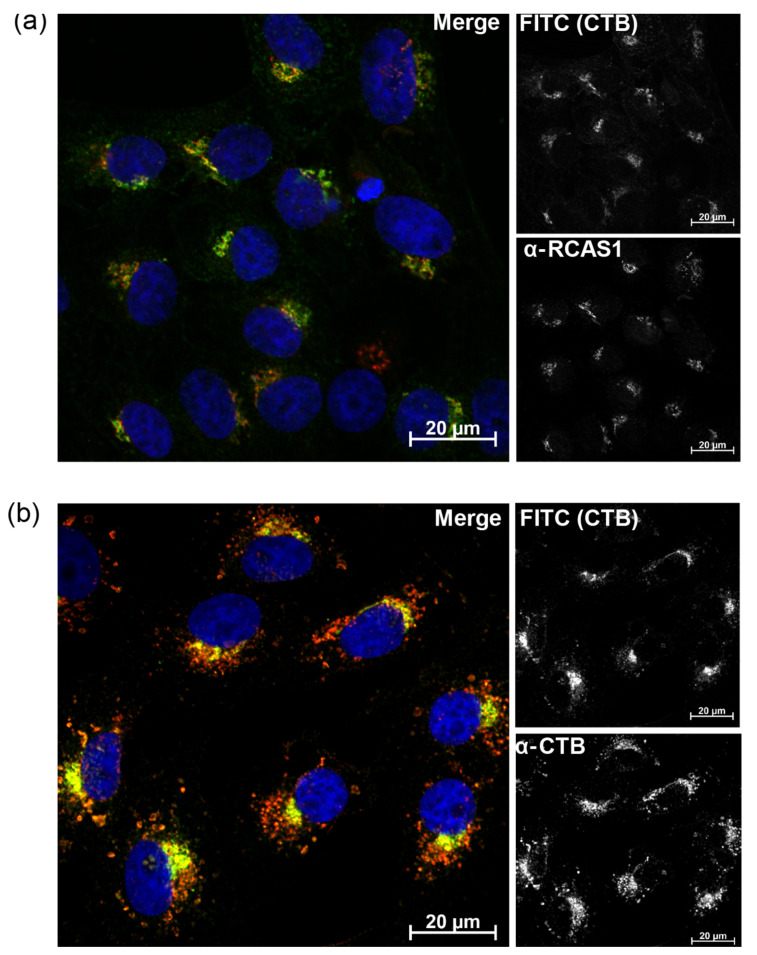
Imaging subcellular localization of FITC-CTB after incubation with Vero cells. Imaging of CTB in Vero cells. (**a**) FITC-CTB (green) was incubated with Vero cells for 24 h before fixation and staining for RCAS1 (red) (**b**) FITC-CTB was incubated with Vero cells for 24 h before fixation and staining for CTB (red). Both images DAPI (blue), colocalised FITC-CTB and α-CTB staining yellow.

While CTB has previously been N-terminally labeled using sortase using flow conditions, the functional consequences of this labeling on trafficking have not been assessed [60]. Earlier studies of the cholera toxin-HRP conjugate using negative stain electron microscopy have shown accumulation of the cholera toxin in all of the cisternae of the Golgi [38]. Unlike the N-terminus of the A2 subunit, the N-terminus of CTB is adjacent to the GM1 binding site, and it was, therefore, possible that the addition of the eight amino-acid linker and FITC might have an adverse effect on either ligand binding or cell entry. We assayed GM1 ganglioside binding affinity and stoichiometry using ITC (Figure S1 and Table 1). GGG-CTB bound GM1 in a 1:1 ratio consistent with wt-CTB, while the labeled FITC-CTB exhibited stoichiometric binding but with a small reduction in affinity. We next investigated the effect of labeling on cell trafficking by the toxin. For CTB, we used Vero (monkey kidney epithelial) cells as a convenient system to study endocytosis, these are functionally equivalent to B-SC-1 cells. After 2 h incubation, the labeled protein was localized to a single region of punctate spots consistent with localization to the Golgi (Figure 6a), as shown by the presence of staining by α-RCAS1 antibodies with a similar morphology [61]. To confirm that this labeling pattern was due to intact FITC-CTB and that peptidic cleavage of the linker sequence had not occurred to release FITC, we carried out immunofluorescent co-staining with an anti-CTB antibody and confirmed that the FITC-CTB showed the same pattern of labeling as the α-CTB antibody (Figure 6b). This pattern of perinuclear labeling is similar to that observed for both.

**Figure 7:**
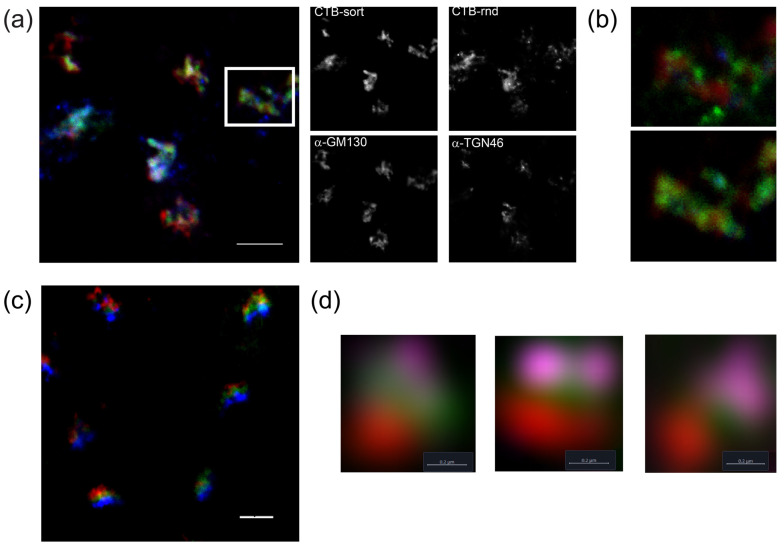
Sortase-labeled CTB is localized to the *medial* Golgi in a COG-independent fashion. (**a**) CTB-Sort localization to the Golgi is less diffuse than randomly labeled CTB. HEK293 cells incubated with both specifically labeled FITC-CTB (green) and CTB randomly labeled with Alexa Fluor 647 (blue) for 30 min before continued growth in media for 2.5 hrs, fixation and staining for GM130 (red) and TGN46 (purple) Scale bar 5 μm. (**b**) Close-up of localization in white box shown in (**a**). Top overlay of CTB-rand (green), GM130 (red) and TGN46 (blue). Bottom overlay of CTB-sort (green) with the same markers. (**c**) FITC-CTB (green) incubated with GM1-supplemented HEK293 COG4 knockout cells before growth in fresh medium for 24 h fixation and staining for GM130 (red) and TGN46 (blue). (**d**) Airyscan imaging of HEK293 cells after incubation with FITC-CTB (green), followed by growth for 2.5 h (left) and 24 h (center and right), treatment with nocodazole for 3 h, fixation and staining for GM130 (red) and TGN46 (violet). Scalebar 0.2 μm.

Endocytosis of the intact cholera toxin has been shown to be mediated by a wide variety of potential pathways, and it is thought to be translocated to the ER via the *trans*-Golgi network [10]. We, therefore, investigated both the time taken to establish the stable labeling pattern and the precise location of the protein in both Vero and HEK293 (human embryonic kidney) cells. In Vero cells, FITC-CTB could be detected as dispersed puncta throughout the cell after 5 min of uptake in both wide-field and confocal imaging; after approximately 30 min, localization to the same distinct perinuclear region as observed for the A2-B_5_ toxoid is apparent, and this localization is essentially complete after approximately 1 h (Figure S2). Co-staining with an antibody to RCAS1, a *trans*-Golgi marker, showed some colocalization, suggesting that the protein is largely resident in a Golgi compartment. Continued cell growth had no effect on the distribution of the protein. In the human cells, a similar pattern was observed (see Figure 7a). Importantly, no ER staining was detected despite using the same pulse-chase approach as utilized with A2-B_5_. The morphology of the labeling pattern in HEK293 cells was again consistent with the labeling of the Golgi, and we, therefore, used immunostaining to identify the compartment. The labeled portion of the cell is again consistently between the GM130 *cis*-Golgi marker and the TGN46 TGN marker (Figure 7a), suggesting the protein is localized to either the *medial*- or *trans*-Golgi. We, therefore, used nocodazole treatment to induce the formation of Golgi mini-stacks [62], which allowed the more precise localization of CTB relative to the *cis*-Golgi and TGN markers (Figure 7d). Following the addition of CTB construct for 30 min, the cells were incubated in a growth medium for 2.5 or 24 h, followed by a 3 h treatment with 5 μM nocodazole before cell fixation and imaging by immunofluorescence (Figure 7d). The signal for FITC-CTB is always observed between that for the *trans*- (TGN46, violet) and *cis*-Golgi (GM130, red) markers consistent with a *medial*-Golgi localization.

How CTB segregates to this compartment is not known. To test whether the protein localization is an artifact of the labeling position, we generated randomly labeled CTB using an AF647-activated ester. This protein sample shows the same localization pattern as the sortase-labeled CTB, though the pattern of the localization is potentially less well-defined, suggesting that random labeling may affect the epitope in CTB required for the observed localization (Figure 7b). Correct sorting of proteins within the Golgi is dependent upon the function of the COG (conserved oligomeric Golgi) complex [63]. This multi-subunit assembly mediates vesicle targeting in retrograde transport pathways, therefore ensuring that the enzymes associated with post-translational modification in the Golgi are segregated to the correct cisternae [64,65]. Defects in seven of the eight COG subunits are associated with congenital disorders of glycosylation due to loss of functional protein [66]. A CRISPR/Cas9 approach has recently been used to generate a set of HEK293 cell lines in which each COG subunit was selectively deleted [67]. These cells are no longer capable of mediating CTB uptake, this is consistent with the key biosynthetic enzymes for the GM1 ganglioside no longer being correctly maintained in the Golgi by COG-mediated retrograde transport. In cell types lacking GM1, it is possible to induce CTB endocytosis by the addition of GM1-ganglioside to cell culture. We used this approach for the COG4 knock out HEK293 cell line to investigate whether the COG complex was also required for CTB transport to the *medial*-Golgi. In all cases, the addition of GM1 to the cell culture restored CTB uptake, and the final distribution of protein within the cell was essentially identical to that observed in the parental HEK293 cell line (Figure 7c). The time taken to establish this localization pattern was, however, delayed, confirming that the COG complex is not essential for retrograde transport of the toxoid but required for efficient transport.

## 3. Conclusions

In conclusion, we have generated two forms of labeled cholera toxoids using sortase-mediated labeling to generate quantitatively labeled proteins. While the A2-B_5_ toxoid shows a mixed pattern of localization between the ER and Golgi, presumably due to KDEL-related shuttling, the B_5_ toxoid largely accumulates in the *medial*-Golgi. Although some protein is present in the *cis*- and *trans*-Golgi, this pattern is more specific than was expected based on previous studies [38]. This localization is not reliant upon the major protein complex controlling retrograde trafficking of proteins in the Golgi though the efficiency is decreased in its absence. Although the origin of this localization is unclear, it suggests that the CTB toxoid has further potential applications in both live cell imaging and in controlled delivery of proteins or other small molecules to the *medial*-Golgi.

## 4. Materials and Methods

**Protein overexpression and purification** MBP-A2-B_5_ was overexpressed from vector pSAB2.1 in *E. coli* C41 (DE3) cells. The complex was isolated from the growth media by sequential amylose affinity, Ni-NTA affinity [68] and size-exclusion chromatography. CTB H74A and GGG-CTB were overexpressed from vectors pSAB2.2 and pSAB2.2(GGG-CTB) in *E. coli* C41 (DE3) cells and purified by sequential Ni-NTA affinity and size-exclusion chromatography. MBP-TEV was overexpressed from vector pMalc5x(TEV) in *E. coli* BL21-Gold (DE3), purified by amylose-affinity chromatography (eluting with 1M glucose) and size-exclusion chromatography. WT-SrtA was overexpressed from vector pET28a(Srt) as described previously [56]. Srt7M was overexpressed from vector pET30b-7M SrtA (Addgene #51141) in *E. coli* BL21-Gold (DE3) and purified by sequential Ni-NTA affinity and size-exclusion chromatography.

**Protein labeling** Dansyl and FITC-depsipeptides were synthesized using the Fmoc strategy for solid-phase peptide synthesis as previously reported. The CTA2-AB_5_ complex (44 μM) was incubated with 1.5 equivalents of dansyl depsipeptide (71 µM) and 10 mol% SrtA (4.4 μM) at 37 °C in 50 mM HEPES, 150 mM NaCl, 5 mM CaCl2 pH 7.5 for 3 h (Figure S10). The same buffer was used for FITC-depsipeptide labeling of both CTB and A2-B5 using SrtA. For labeling using Srt7M, a Ca-free buffer was used. Individual protocols for protein labeling are described in the supplementary information.

**Cell culture and fluorescence imaging** Vero, BSC-1 and HEK293T cells were cultured using standard protocols in Dulbecco’s Modified Eagle Medium supplemented with 10% fetal bovine serum and glutamine. For the culture of HEK293T cells, poly-lysine coated slides were used. Toxoids were added to cells and incubated in a complete medium prior to fixation using either paraformaldehyde (Vero and HEK293T) or methanol (BSC-1). Cell membrane-bound proteins were removed by acid-washing. For imaging of Golgi mini-stacks, cells were incubated with 5 μM nocodazole for 3 h prior to fixation. Cells were imaged using an Axio Imager Z2 LSM880 confocal microscope (Vero cells), an Improvision 3DM wide-field imaging system (BSC-1 and HEK293T) and a LSM 880 AiryScan microscope (HEK293T). Full details of cell culture and imaging can be found in the supplementary information.

## Figures and Tables

**Table 1:** Thermodynamic parameters for CTB binding to GM1 ganglioside determined by isothermal titration calorimetry.

Title 1	N	*K*_d_/nM	ΔG/kJ mol^−1^	ΔH/kJ mol^−1^
WT-CTB	0.93	60 ± 20	−41.2 ± 0.9	−57.7 ± 1.7
GGG-CTB	0.93	90 ± 20	−40.2 ± 0.5	−58.2 ± 2.1
FITC-CTB	1.08	358 ± 62	−36.7 ± 0.4	−69.0 ± 4.6

## Data Availability

The data presented in this study will be available from the University of Leeds Data Repository.

## Supplementary Materials

The following supporting information can be downloaded at: https://www.mdpi.com/article/10.3390/toxins16040194/s1, Figure S1: ITC of GM1os titrated into wt-CTB, GGG-CTB and FITC-CTB; Figure S2: Time course of Vero cell incubation with FITC-CTB; Figure S3: Analytical data for purification of MBP-tagged TEV by size-exclusion chromatography; Figure S4: Structure of the expression construct for MBP-A2-B_5_; Figure S5: Structure of the expression construct for MBP-A2-B_5_; Figure S6: Mass spectrum of the truncated CTA2-B_5_ complex; Figure S7: Structure of the expression construct for GGG-CTB; Figure S8: Purification and analysis of GGG-CTB; Figure S9: Purification of sortase 7M for use in labeling reactions; Figure S10: Test labeling of A2-B_5_ complex analyzed by ESMS.
