# Supplementary material for: Sortase-Modified Cholera Toxoids Show Specific Golgi Localization
Source: Toxins (Basel). 2024 Apr 16;16(4):194. doi: 10.3390/toxins16040194 (PMC11054894; doi:10.3390/toxins16040194)
Supplement: Supplementary file 1 [file toxins-16-00194-s001.zip › toxins-2882772-supplementary.pdf]

Supplementary Materials: Sortase-Modified Cholera Toxoids Show Specific Golgi Localization

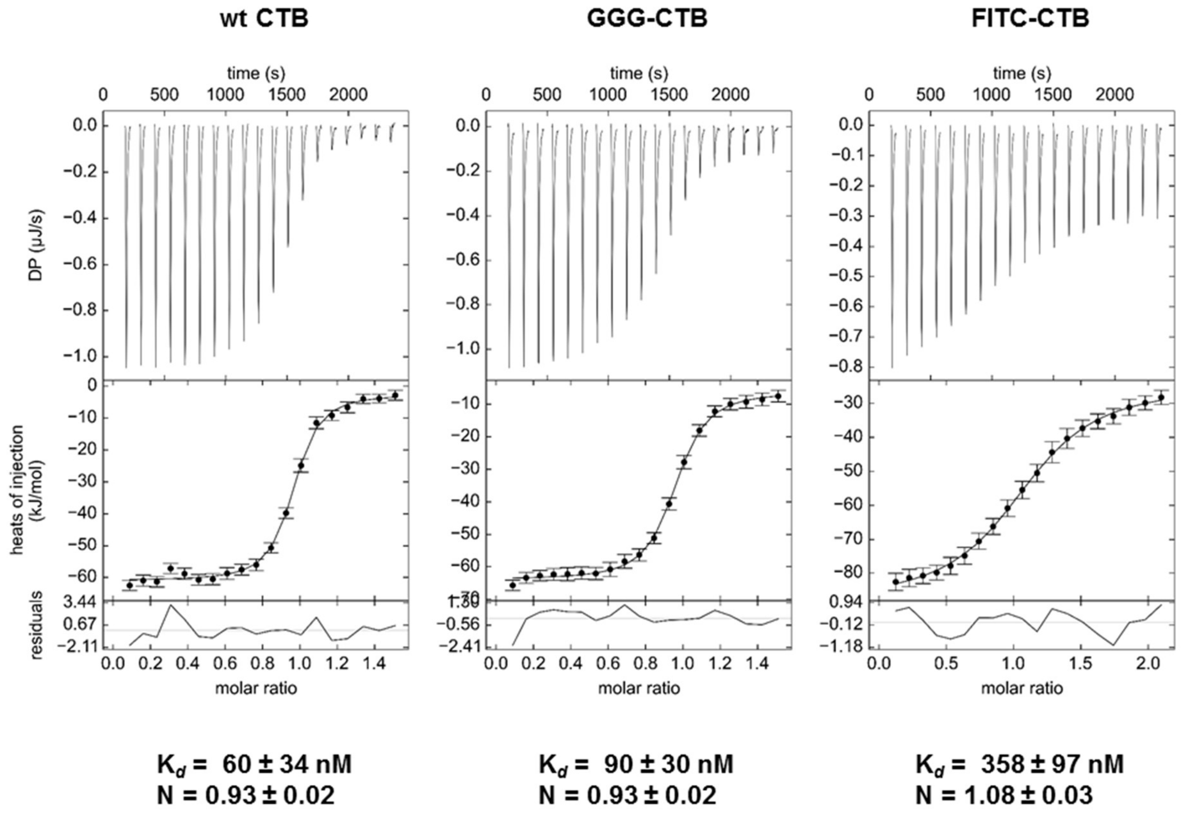

Figure S1 ITC of GM1os titrated into wt CTB, GGG-CTB and FITC-CTB Titration of GM1os into wt CTB (left), GGG-CTB (centre), and FITC-CTB (right). Each plot shows the baseline-adjusted raw thermogram (upper), the fitted integral data (middle) and the residual error of the fitting (lower).

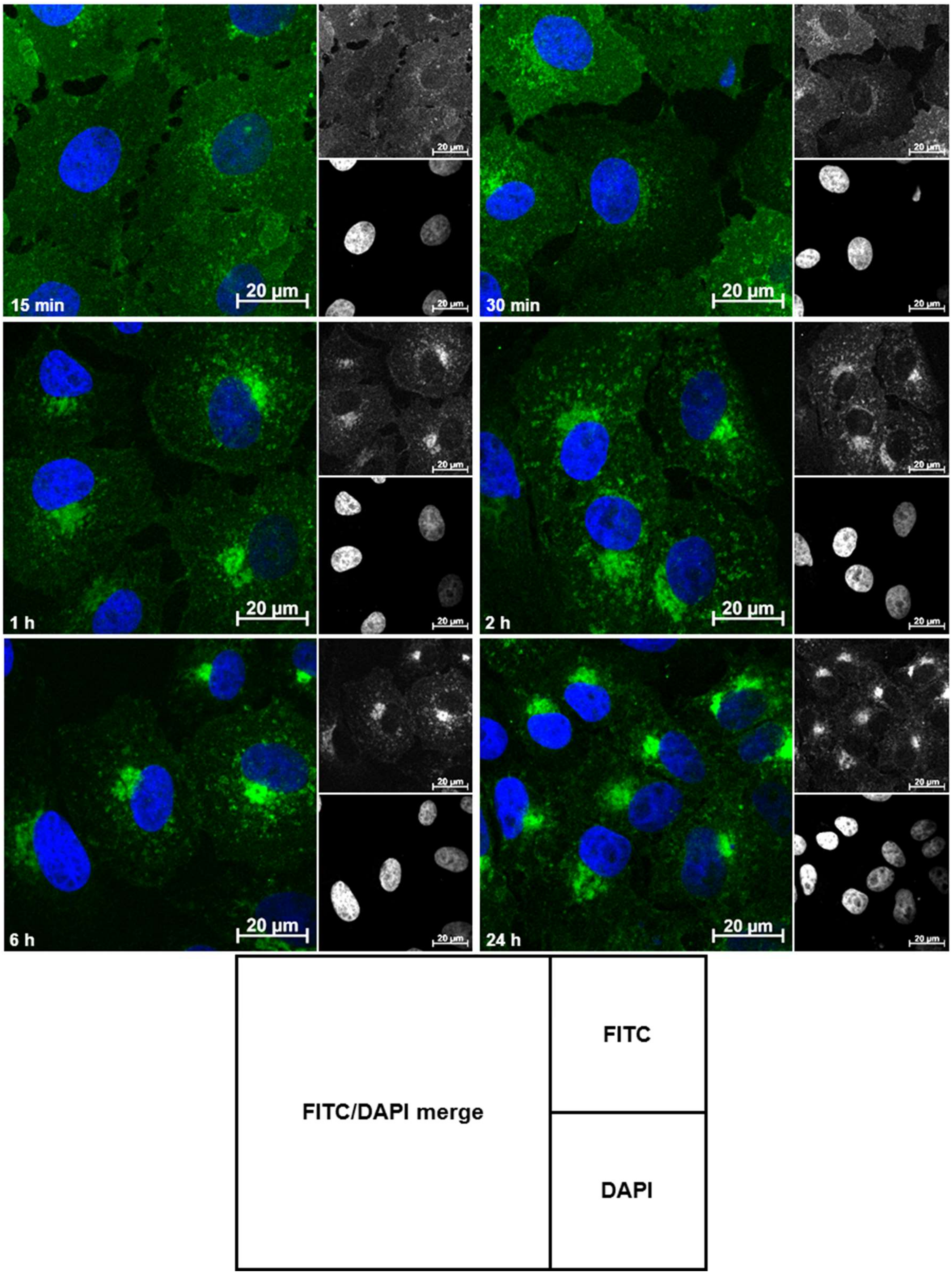

Figure S2. Time course of Vero cell incubation with FITC-CTB LSCM imaging of Vero cells incubated with FITC-CTB (2 µg mL^{-1} protein) and fixed after 15 min, 30 min, 1 h, 2 h, 6 h or 24 h. Each panel shows a false-colour merged image (left) of FITC (CTB; green) and DAPI (nuclei; blue), in addition to individual green (FITC; upper right) and blue (DAPI; lower right) channels.

Overexpression and purification of proteins

MBP-TEV (maltose binding protein-tobacco etch virus protease fusion).

MBP-TEV was overexpressed as an uncleavable MBP fusion protein. The coding sequence for TEV was originally obtained as a TEV-autocleavable MBP-His-TEV fusion (pMAL-C2) from Dr. T. Edwards (Univ of Leeds). The coding sequence for TEV was amplified by PCR and subcloned into pMalc5x using *BamHI* and *PstI.* A single colony of *E. coli* BL21-Gold (DE3) cells harbouring the pMAL-MBP-TEV plasmid was used to inoculate LB media (5 mL, 100 µg/mL ampicillin). The starter culture was incubated at 37 °C for 18 h before 3 ml was added to LB media (1 L, 100 µg/mL ampicillin), which was then grown at 37 °C. Protein overexpression was induced once the OD_{600} had reached ~ 0.7 with the addition IPTG (1ml, 500 mM). Incubation was continued for 20 h at 20 °C before the cells were isolated by centrifugation (10 × k*g*, 10 min), the supernatant discarded and the cell pellet retained.

The frozen bacteria pellet was resuspended in 50 mM Tris (pH 8.0) 1 mM β-mercaptoethanol *(*10 mL) at 4 °C. The suspension was mechanically disrupted using a Constant Systems Cell Disruptor (20 kpsi, 10 mL injections, 4 °C), the lysate cleared by centrifugation (30 × kg, 45 minutes), the pellet discarded and the supernatant passed down a amylose affinity column (Qiagen, 20 ml) equilibrated in 50 mM Tris pH 8.0 1 mM β-mercaptoethanol The column was washed with the same buffer (5 × CV) and the protein eluted with 50 mM Tris (pH 8.0), 150 mM NaCl, 1 mM β-mercaptoethanol containing 10 mM Maltose (5 × CV). Protein-containing fractions were identified by SDS-PAGE and concentrated to a total volume of 1 mL by centrifugal concentration at 4 °C. The protein solution was applied to a size-exclusion column and isocratically eluted into 50 mM Tris (pH 8.0), 150 mM NaCl, 1 mM β-mercaptoethanol before the purity of the isolated protein was analysed by SDS-PAGE. The protein concentration was measured by UV spectroscopy at 280 nm using a theoretical extinction molar coefficient of 98329 M^{-1} cm^{-1}

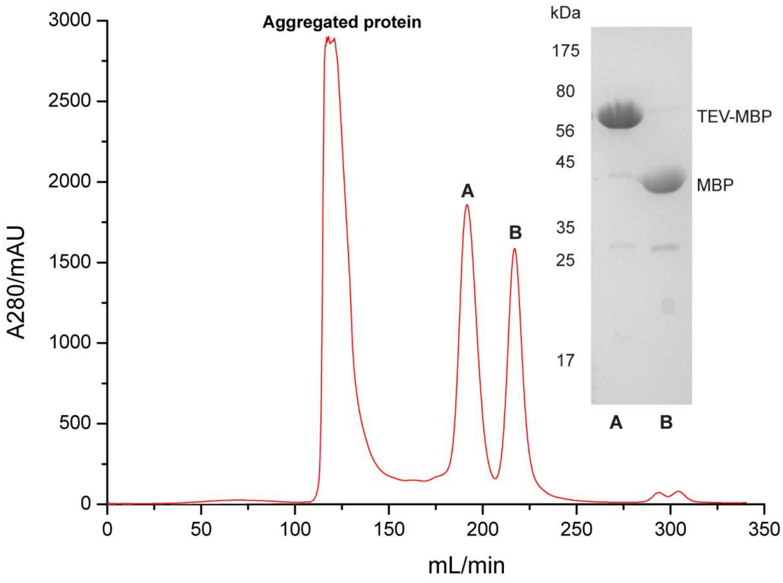

Figure S3 Analytical data for purification of MBP-tagged TEV by size-exclusion chromatography.

MBP-A2-B$_5$ MBP-A2-B$_5$ was produced using plasmid pSAB2.1 which was based on a synthetic gene ordered from Genscript for co-expression of maltose-binding protein-A2 and a T1A mutant of CTB (El Tor strain) subcloned into pMalp5x between the NdeI and PstI sites. pSAB2.1 includes a tobacco etch virus (TEV) protease recognition sequence followed by a triglycine linker and the El Tor cholera toxin A2 domain (residues 200-240). This is followed by a ribosome binding site, an *E. coli* heat labile toxin IIb periplasmic targeting sequence and the El Tor CTB sequence encoding a T1A mutation (out of frame with the MBP-A2 sequence to allow polycistronic expression of the genes).

```
aggatttcacatatgggatccgaaaacctgtactttcagggtggcggtgatgaaaaaacc
 R  I  S  H  M  G  S  E  N  L  Y  F  Q  G  G  G  D  E  K  T
caaagtcatggtgtaaaattccttgacgaataccaatctaaagttaaaagacaaatattt
 Q  S  H  G  V  K  F  L  D  E  Y  Q  S  K  V  K  R  Q  I  F
tcaggctatcaatctgatattgatacacataatagaattaaggatgaattatgacctcgag
 S  G  Y  Q  S  D  I  D  T  H  N  R  I  K  D  E  L  -
gtgaattcacgagcaattgaccaacaaggaccatagattatgagctttaagaaaattatc
                                       M  S  F  K  K  I  I
aaggcatttgttatcatggctgctttggtatctgttcaggcgcatgcagctcctcaaaat
 K  A  F  V  I  M  A  A  L  V  S  V  Q  A  H  A  A  P  Q  N
attactgatttgtgcgcagaataccacaacacacaaatatatacgctaaatgataagatc
 I  T  D  L  C  A  E  Y  H  N  T  Q  I  Y  T  L  N  D  K  I
ttttcgtatacagaatcgctagcgggaaaaagagagatggctatcattacttttaagaat
 F  S  Y  T  E  S  L  A  G  K  R  E  M  A  I  I  T  F  K  N
ggtgcaattttcaagtagaggtaccaggtagtcaacatatagattcacaaaaaaagcg
 G  A  I  F  Q  V  E  V  P  G  S  Q  H  I  D  S  Q  K  K  A
attgaaaggatgaaggataccctgaggattgcatatcttactgaagctaaagtcgaaaag
 I  E  R  M  K  D  T  L  R  I  A  Y  L  T  E  A  K  V  E  K
ttatgtgtatggaataataaaacgcctcatgcgatcgccgcaattagtatggcaaactaa
 L  C  V  W  N  N  K  T  P  H  A  I  A  A  I  S  M  A  N -
gttttccctgcag
```

Figure S4 Structure of the expression construct for MBP-A2-B5. The coding sequence for the El Tor ChTx A2 peptide (green)is inserted in frame with malE in the pMalp5x backbone down stream of Factor Xa (grey) and TEV (khaki) cleavage sequences. The coding sequence for the El Tor ChTx T1A CTB mutant (red) is incorporated downstream of the *E. coli* heat-labile toxin IIb periplasmic targetting sequence (pink).

The MBP-CTA2 fusion protein was co-expressed with CTB in *E. coli* C41 cells harbouring the pSAB 2.1 plasmid. Co-expressing the proteins triggered self-assembly of an MBP-CTA2/B$_5$ (MBP-AB$_5$) complex in the periplasm of the cells. A combination of amylose and nickel affinity chromatography was used to isolate the MBP-AB$_5$ protein complex. Initially, amylose affinity chromatography captured any species containing MBP, including the MBP-AB$_5$ and uncomplexed MBP-CTA2 proteins. A nickel affinity column was used to separate out the resulting mixture of proteins; the pentameric B-subunit of the MBP-AB$_5$ complex bound to the nickel resin while the remaining MBP-CTA2 protein passed through the column. After purification the protein can be seen as a discrete band on an SDS-PAGE gel.. However, the hexamer appears to be unstable under the detergent conditions of SDS-PAGE analysis and it can be seen to partially dissociate on the gel into pentameric CTB and MBP-CTA2 proteins. If

the MBP-AB_5 protein is completely denatured by heating before applying to the gels, it falls apart into its constituent CTB monomer and MBP-CTA2 proteins

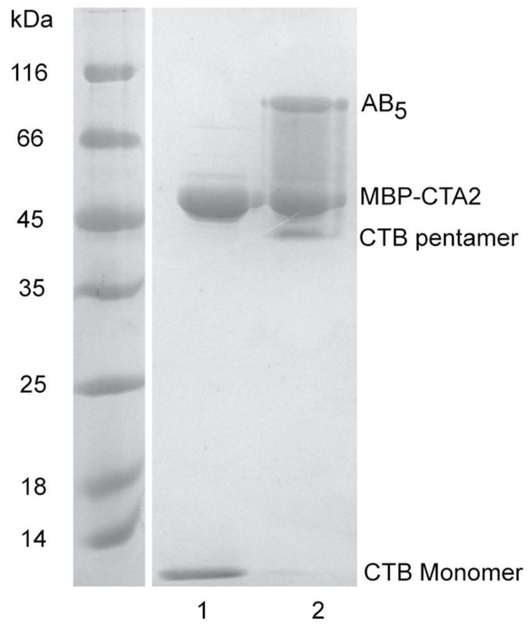

Figure S5 Structure of the expression construct for MBP-A2-B5. The coding sequence for the El Tor ChTx A2 peptide (green)is inserted in frame with malE in the pMalp5x backbone down stream of Factor Xa (grey) and TEV (khaki) cleavage sequences. The coding sequence for the El Tor ChTx T1A CTB mutant (red) is incorporated downstream of the *E. coli* heat-labile toxin IIb periplasmic targetting sequence (pink).

Preparation of the AB_5 complex for SrtA-mediated ligation

The MBP-purification tag had to be removed from the AB_5 complex in preparation for SrtA-mediated modification. The MBP-CTA2 construct had been designed to include an optimum TEV protease recognition sequence (ENLYFQG) to allow enzymatic cleavage and separation of the two proteins. TEV protease will specifically hydrolyse the amide bond between the QG amino acid residues to expose a triglycine sequence at the *N*-terminus of the CTA2-peptide.

TEV protease-mediated MBP cleavage

Treating the MBP-AB_5 complex (45 μM) with 10 mol% MBP-TEV (4.5 μM) for 1 hour at room temperature successfully generated a truncated CTA2-AB_5 complex. The reaction was monitored by ESMS and the molecular weight of the MBP-CTA2 protein decreased by the mass of the CTA2-peptide, 5055 Da. However, at higher MBP-TEV concentrations or longer incubation periods, indiscriminate proteolysis of the protein was observed. The truncated AB_5 complex was isolated from the cleavage mixture using nickel affinity chromatography, and two sets of signals corresponding to the CTA2 and CTB chains were identified by ESMS (Figures S6).

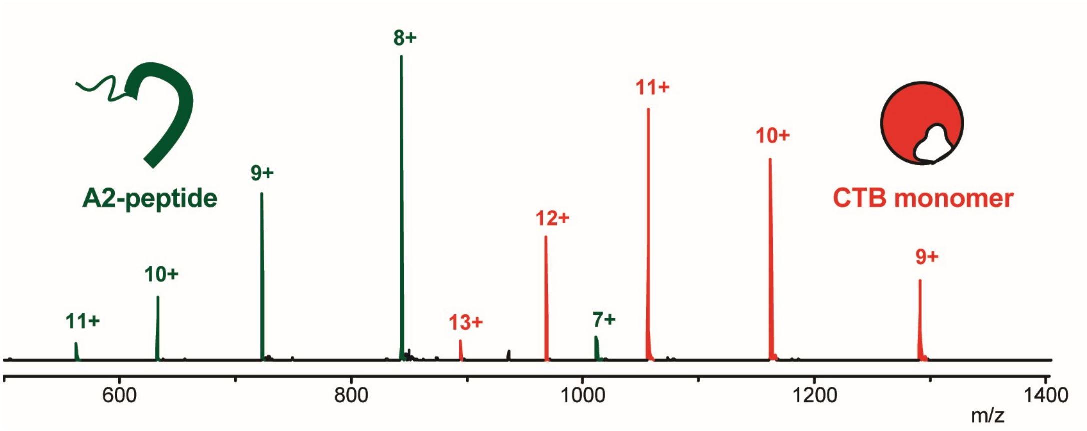

Figures S6: Mass spectrum of the truncated CTA2-AB_5 complex. Green: CTA2 peptide (5055 Da); Red: CTB monomer (11611 Da).

GGG-CTB The expression construct for GGG-CTB was based upon the expression constructs for CTB used in Ross et al.., 2019. Vector pSAB2.2 was generated by excision of the coding sequence for MBP-A2 using dual MfeI sites. The coding sequence for GGG-CTB was generated by assembly PCR using internal primers A1-F1 and terminating primers FT and RT to generate a 350 bp fragment which was cloned into pSAB2.2 using SphI and PstI.

A1: 5'-CTGTTCAGGCGCATGCAGGAGGTGGCACTCCTCAAAATATTACTGATTTGTGCGCAGAAT ACCACAACAC-3'

B1: 5'-CTCTTTTTCCCGCTAGCGATTCTGTATACGAAAAGATCTTATCATTTAGCGTATATATTTGT GTGTTGTGGTATTCTGCG-3'

C1: 5'-GCTAGCGGGAAAAAGAGAGATGGCTATCATTACTTTTAAGAATGGTGCAATTTTTCAAGTA GAGGTACCAGGTAGTC-3'

D1: 5'-GCAATCCTCAGGGTATCCTTCATACGTTCGATTGCCTTTTTTTGTGAATCTATATGTTGACT ACCTGGTACCTCTACTTG-3'

E1: 5'-GATACCCTGAGGATTGCATATCTTACTGAAGCTAAAGTCGAAAAGTTATGTGTATGGAATA ATAAA-3'

F1: 5'-CCTGCAGGGAAAACTTAGTTTGCCATACTAATTGCGGCGATCGCATGAGGCGTTTTATTAT TCCATACACATAA-3'

FT: 5'-CATGCGCCTGAACAG-3'

RT: 5'-CCTGCAGGGAAAACTTAG-3'

```
acaggaaacagccagtccgtttaggtgttttcacgagcaattgaccaacaaggaccatagattatg
                                                                 M
agctttaagaaaattatcaaggcatttgttatcatggctgctttggtatctgttcaggcgcatgca
 S  F  K  K  I  I  K  A  F  V  I  M  A  A  L  V  S  V  Q  A  H  A
ggaggtggcactcctcaaaatattactgatttgtgcgcagaataccacaacacacaaatatatacg
 G  G  G  T  P  Q  N  I  T  D  L  C  A  E  Y  H  N  T  Q  I  Y  T
ctaaatgataagatcttttcgtatacagaatcgctagcgggaaaaagagagatggctatcattact
 L  N  D  K  I  F  S  Y  T  E  S  L  A  G  K  R  E  M  A  I  I  T
tttaagaatggtgcaatttttcaagtagaggtaccaggtagtcaacatatagattcacaaaaaaa
 F  K  N  G  A  I  F  Q  V  E  V  P  G  S  Q  H  I  D  S  Q  K  K
gcgattgaaaggatgaaggataccctgaggattgcatatcttactgaagctaaagtcgaaaagtta
 A  I  E  R  M  K  D  T  L  R  I  A  Y  L  T  E  A  K  V  E  K  L
tgtgtatggaataataaaacgcctcatgcgatcgccgcaattagtatggcaaactaagttttccct
C  V  W  N  N  K  T  P  Q  A  I  A  A  I  S  M  A  N  -
gcag
```

Figure S7 Structure of the expression construct for GGG-CTB. The coding sequence for the periplasmic targetting sequence of the *E. coli* heat labile toxin IIb is followed by El Tor ChTx B subunit between the MfeI and PstI sites of the pMalp5x backbone.

Overexpression/purification of GGG-CTB

GGG-CTB was overexpressed in *E. coli* C41(DE3) grown in LB media by IPTG (0.5 mM) induction at 25 °C overnight. The overexpressed protein was isolated from the growth medium by precipitation with saturating ammonium sulfate and purified by Ni^{2-}affinity chromatography and size-exclusion chromatography (Figure S8). The identity of the protein was confirmed by LC-MS; a protomer mass of 11814.0 Da was detected (11,813.8 Da calculated). Approximately 5 mg pure protein was isolated per 1 L cultured cells.

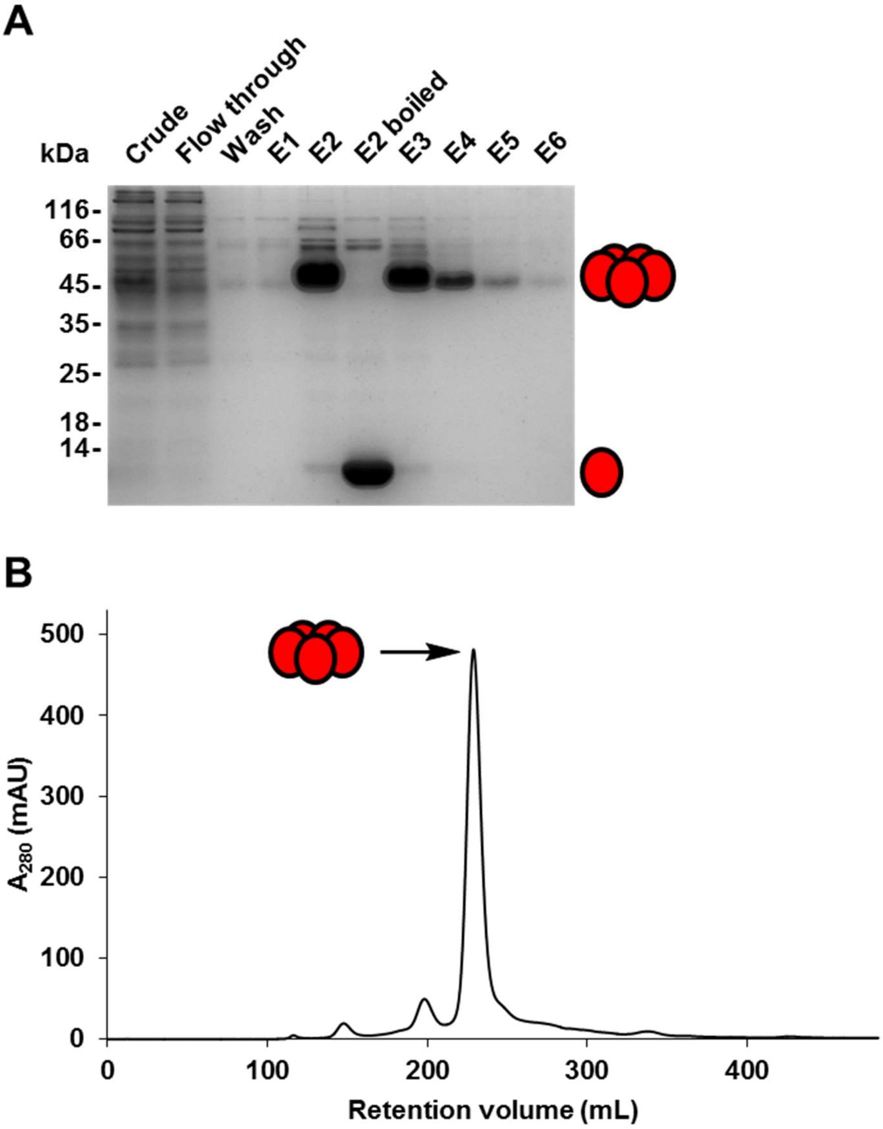

Figure S8 Purification and analysis of GGG-CTB A. SDS-PAGE analysis of GGG-CTB purification by Ni^{2+} affinity chromatography. GGG-CTB as pentamer and dissociated protomer are indicated. B. A_{280} trace from SEC purification of Ni^{2+}-purified GGG-CTB, with the desired protein peak indicated.

Overexpression purification of Srt7M

The sortase 7M plasmid was obtained from Hidde Ploegh and provided by Addgene (plasmid #51141), it was transformed into *E. coli* BL21 gold (DE3) before being expressed in auto-induction media (AIM) at 30 °C for 5 hours before being left at 25 °C overnight. Following cell lysis, the sortase 7M was purified using Ni-NTA and size-exclusion chromatography using a Superdex® S75 column run in filtered Tris-buffered saline at pH 7.4

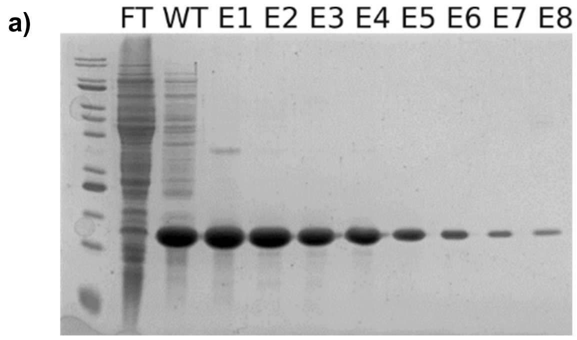

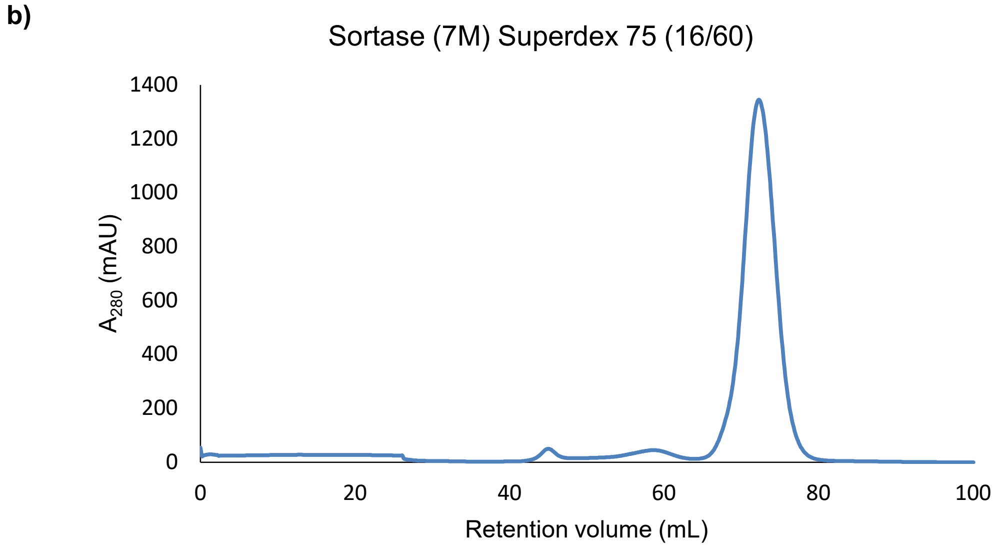

Figure S9 Purification of sortase 7M for use in labelling reactions a) SDS-PAGE gel of Ni-NTA column flow through (FT), wash (WT) and elution fractions (E1-8). b) SEC trace for Srt 7M using Superdex® S75.

SrtA-mediated protein labelling

Labelling of A2-B$_5$ and CTB using WT sortase

The CTA2-AB$_5$ complex (44 μM) was incubated with 1.5 equivalents of dansyl depsipeptide **2.37** (71 μM) and 10 mol% SrtA (4.4 μM) at 37 °C in 50 mM HEPES, 150 mM NaCl, 5 mM CaCl2 pH 7.5 for 3 hours (Figure S10). The same buffer was used for FITC-depsipeptide labelling of both CTB and A2-B5 using SrtA.

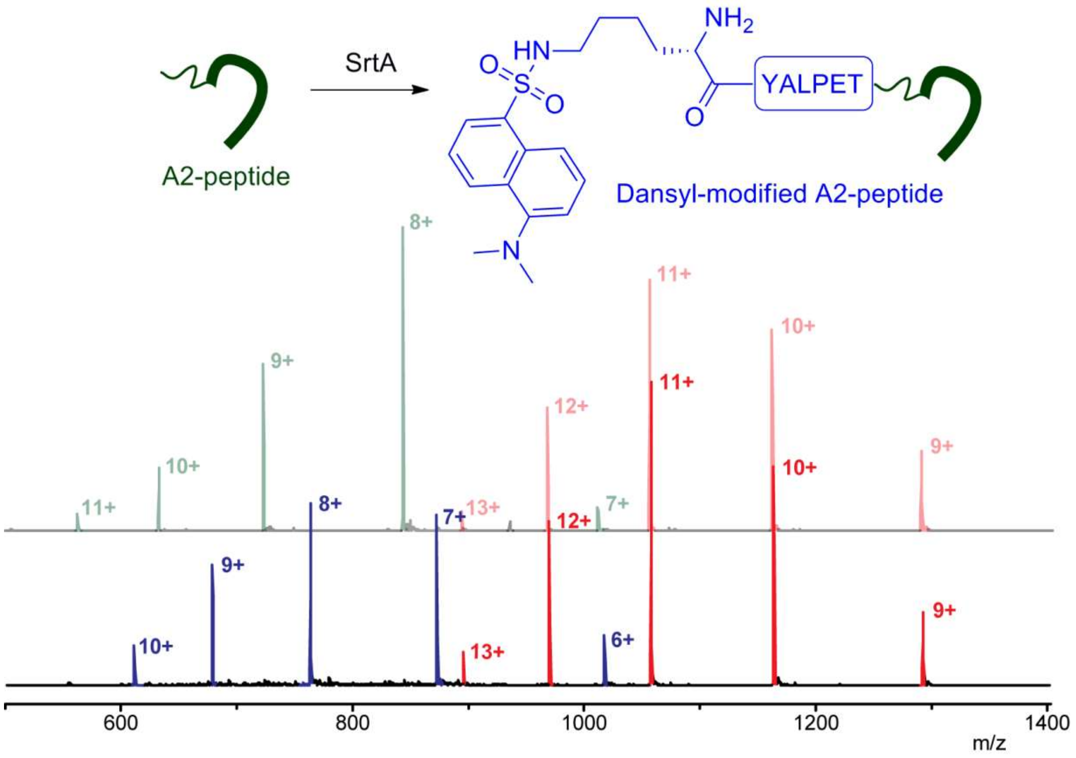

Figure S10 Test labelling of A2-B5 complex analysed by ESMS

Culture and imaging of BSC-1 cells

B-SC-1 kidney cells were grown overnight in MEM medium (Sigma-Aldrich, Poole, UK) supplemented with 10% FBS (Invitrogen, Paisley UK) and 0.292 g/L glutamine on glass coverslips. FITC-conjugated cholera toxin was added to the medium at 10 μg/ml and cells were incubated at 37 °C for the stated periods. The coverslips were then washed for 3 x 30 s with ice-cold 0.5 M glycine at pH 2.2 on ice to remove excess toxin bound to the cell surface prior to fixation. Cells were rinsed with ice-cold phosphate buffered saline (PBS) beforeand after the addition of -20°C methanol for 2 minutes followed by incubation with PBS/ 3% bovine serum albumin. Primary and secondary antibodies were also diluted in PBS/ 3% bovine serum albumin and applied to coverslips for 1 h at room temperature, interspaced with 3 x 15 min PBS washes. Coverslips were then counter-stained with DAPI and mounted onto glass slides with Mowiol mountant. Primary antibodies used were mouse anti-GM130 and mouse anti-Calnexin (BD Transduction Laboratories, Cambridge, UK) Fluorophore-conjugated secondary antibodies used were from Jackson ImmunoResearch Laboratories (West Grove, PA). Immunostained cells were imaged using an Improvision 3DM system (Improvision, Coventry, UK): an IX-81 microscope fitted with an ASi PZ-2000 XYZ stage (Applied Scientific Instruments, Eugene, OR) and a DG-4 illumination system (Sutter, Novato, CA, USA) comprising of filter sets with single-band exciters and multi-band dichroic and emission filters (Semrock, Rochester, NY) was used. A Hamamatsu Orca-ER cooled CCD camera and Volocity version 4.2 (Improvision) were used to capture images. Images were cropped using ImageJ 1.44o.

Culture and imaging of Vero cells

Vero cells were grown in DMEM (containing 4500 mg L^{-1} glucose, L-glutamine, sodium pyruvate, sodium bicarbonate and pyridoxine; Sigma Aldrich) containing 10% (*v/v*) foetal bovine serum (FBS; Lonza) and 250 U mL^{-1} (each) penicillin/streptomycin (pen/strep; Lonza) at 37 °C with 5% CO_2. For imaging experiments, cells were seeded (1×10^5 mL^{-1}) into multi-well plates containing coverslips.and cells growth to 60-80% confluency. The cells were washed with PBS before addition of the protein (2 μg/mL) in DMEM supplemented as above for continued growth. Cells were fixed by removal of growh media, washing with 2 × 1 mL PBS and addition of paraformaldehyde (4% v/v) in PBS for ten min at room temperature. Cells were washed with 2 × 1 mL PBS and stored at 4 °C. Cell membranes were permeabilised with 0.5 mL Triton-X 100 (0.5% *v/v*) in PBS for 5 min at room temperature. Cells were washed with 3 × 1 mL PBS for 5 min at room temperature, and non-specific protein binding was blocked by treatment with blocking buffer (1% BSA (*w/v*), 300 mM glycine in PBST) for 1 h at room temperature. Blocking buffer was removed and cells treated with primary antibody diluted in 1% (*w/v*) BSA in PBST at 4 °C overnight. Cells were washed with 3 × 1 mL PBS for 5 min and treated with secondary antibody diluted in 1% (*w/v*) BSA in PBST for 1 h at room temperature. Antibody details and staining conditions are described below.

Coverslips were mounted on microscope slides using 12 μL (12-well plate) or 6 μL (24 –well plate) ProLong Gold antifade mountant with DAPI (Invitrogen), set overnight in darkness at room temperature, and sealed with nail varnish.Mounted cells were visualised with an Axio Imager Z2 LSM880 upright confocal microscope (Zeiss) equipped with Diode 405 m, Argon 458/488/514 nm, DPSS 561 nm and HeNe 633 nm lasers and a GaAsp detector with fully tunable emission detection. Emission filter parameters for multiple fluorophore imaging were designed using the Fluorescence SpectraViewer tool (Thermo Scientific). Images were processed using Zen lite 2.3 software (Zeiss).

**Recognises**	**Species**	**Supplier**	**Dilution**	**Incubation time**	**Incubation temperature (°C)**
CTB	Rabbit	Sigma Aldrich (C3062)	10,000	Overnight	4
RCAS1 (Golgi)	Rabbit	Cell Signalling Technology (D2B6N)	100	Overnight	4
Rabbit (CTB) Alexa Fluor 555	Donkey	Invitrogen (A-31572)	1000	1	Room
Rabbit (RCAS1) Alexa Fluor 594	Chicken	Invitrogen (A-21442)	500	1	Room

Culture and imaging of HEK293T cells

HEK293T cells or COG4 KO cells were seeded onto poly-lysine coated coverslips in DMEM media supplemented with 10% FBS, 1% pen/strep solution and 1% Glutamax (all Invitrogen) The media was supplemented with 3.2 μM Ganglioside GM1 as required. Fluorescently labelled protein (2 μg/mL) was added and cells for the indicated time prior to fixation. Cells were fixed by washing twice with PBS preior to addition of 4% paraformaldehyde in PBS for 15 min. Cells were washed twice with PBS followed by 20 mM glycine in PBS before storage in 0.02% NaN_3 in PBS at 4 °as required. For nocodazole treatment 5 mM nocodazole in DMEM was added to cells followed by incubation at 37 °C for 3 hours prior to fixation.

Cells were permeabilised in 0.1% (w/v) Saponin, 2% (w/v) BSA in 20 mM glycine in PBS for 30 min at room temperature before addition of primary antibodies in 20 mM glycine in PBS for 1 hour, washing and addition of secondary antibodies in 20 mM glycine in PBS. Slide were mounted with ProLong(R) Gold Antifade and imaged using a Zeiss LSM880 with an Airyscan detector using a 63x objective.

***cis*-Golgi marker (conjugated primary and secondary):** Alexa Fluor®647 Mouse anti-GM130, BD Pharmingen™, Cat. 558712.

***trans*-Golgi marker (primary):** TGN46/38, GeneTex, Cat. GTX74290.

***trans*-Golgi marker (secondary):** Donkey anti-Sheep IgG (H+L) Cross-Adsorbed Secondary Antibody, Alexa Fluor™ 568, Invitrogen, Cat. A21099.
